# A novel pyrimidine–guanidine derivative YYH-6 confers tomato resistance to TYLCV via multitarget regulation of host defense genes and proteins

**DOI:** 10.1093/hr/uhag120

**Published:** 2026-04-06

**Authors:** Miao Yu, Kexin Liu, Jing Shu, Jing Yang, Yuhang Yang, Dongyang Liu, Ting Liu, Lianqiang Jiang, Zhiping Wang, Zihao Xia, Mengnan An, Xinghai Li, Yuanhua Wu

**Affiliations:** College of Plant Protection, Shenyang Agricultural University, Shenyang, Liaoning 110866, China; College of Agriculture and Horticulture, Liaoning Agricultural Vocational and Technical College, Yingkou, Liaoning 115000, China; College of Plant Protection, Shenyang Agricultural University, Shenyang, Liaoning 110866, China; College of Plant Protection, Shenyang Agricultural University, Shenyang, Liaoning 110866, China; College of Plant Protection, Shenyang Agricultural University, Shenyang, Liaoning 110866, China; Liangshan Prefecture Branch of Sichuan Tobacco Company, Liangshan, Sichuan 615000, China; Liangshan Prefecture Branch of Sichuan Tobacco Company, Liangshan, Sichuan 615000, China; Liangshan Prefecture Branch of Sichuan Tobacco Company, Liangshan, Sichuan 615000, China; College of Plant Protection, Shenyang Agricultural University, Shenyang, Liaoning 110866, China; College of Plant Protection, Shenyang Agricultural University, Shenyang, Liaoning 110866, China; College of Plant Protection, Shenyang Agricultural University, Shenyang, Liaoning 110866, China; College of Plant Protection, Shenyang Agricultural University, Shenyang, Liaoning 110866, China; College of Plant Protection, Shenyang Agricultural University, Shenyang, Liaoning 110866, China

## Abstract

Tomato yellow leaf curl virus (TYLCV) poses a serious threat to global tomato production. Current management strategies remain limited, highlighting the need for novel antiviral agents. In this study, we evaluated the efficacy of a newly synthesized pyrimidine–guanidine derivative, YYH-6, against TYLCV in tomato (*Solanum lycopersicum* cv. Micro-Tom) and investigated its underlying mechanisms. Foliar application of YYH-6 significantly reduced viral accumulation and alleviated disease symptoms. Transcriptomic analysis revealed that YYH-6 treatment upregulates defense-related pathways such as plant–pathogen interaction, MAPK signaling, and phenylpropanoid biosynthesis. Results of TRV-based virus-induced gene silencing (VIGS) further indicate that *RPP13* and *ALS2* function as important resistance genes induced by YYH-6. Activity-based protein profiling, LC–MS/MS, and reverse genetic verification by VIGS indicate that serine hydroxymethyltransferase and STI1 domain-containing proteins are potential target proteins of YYH-6 for TYLCV inhibition. Our findings demonstrate that YYH-6 suppresses TYLCV infection through multitarget modulation of host defense pathways, making it a promising candidate for sustainable management of tomato viral diseases.

## Introduction

Tomato (*Solanum lycopersicum* L.) is one of the most economically important horticultural crops globally, valued for its nutritional richness and versatile culinary applications. According to FAOSTAT, in 2023, the global harvested area of tomatoes expanded to approximately 5.41 million hectares, with production reaching about 192 million tons and the gross production value amounting to approximately USD 116.087 billion, further underscoring its substantial economic importance. However, tomato production systems worldwide are increasingly threatened by viral diseases, among which tomato yellow leaf curl virus (TYLCV) has emerged as a devastating pathogen that causes severe yield losses and quality deterioration [1].

TYLCV, a species of the genus *Begomovirus* in the family Geminiviridae, is transmitted primarily by the whitefly *Bemisia tabaci* in a persistent, circulative manner [2, 3]. TYLCV was first reported in the Middle East in the 1960s. It has since spread rapidly across continents, invading major tomato-growing regions in Asia, Europe, Africa, and the Americas [4–6]. Upon TYLCV infection, tomato plants exhibit characteristic symptoms including leaf yellowing, curling, stunting, and reduced fruit set. Susceptible tomato varieties can suffer yield losses of up to 100%, making TYLCV a major threat to global tomato production [7]. The threat of TYLCV to sustainable tomato production is further exacerbated by its capacity for rapid evolution, recombination into novel strains, and adaptation to new hosts [8, 9].

Current management of TYLCV integrates chemical control of whiteflies, planting of resistant cultivars, and agronomic practices such as crop rotation and physical barriers [10, 11]. However, these approaches face significant limitations. Excessive use of insecticides not only increases production costs but also causes environmental pollution, development of insecticide resistance in whiteflies, and harm to nontarget organisms including pollinators [12, 13]. Despite the identification and use of major TYLCV resistance genes (*Ty-1*, *Ty-2*, *Ty-3*, *Ty-4*, *ty-5*, and *Ty-6*) in breeding, symptoms of tomato yellow leaf curl disease (TYLCD) sporadically manifest in resistant cultivars, especially under high-temperature stress [14, 15]. In addition, the genetic background of the host plant significantly influences the efficiency and durability of resistance [16]. Current research on anti-TYLCV compounds focuses on screening natural products, developing synthetic chemicals, and identifying plant immunity inducers [17–21]. Several natural substances, such as novel nor-triterpenoids from *Euphorbia resinifera*, eugenol, and gibberellin, have exhibited efficacy against TYLCV [17–19]. Similarly, synthetic compounds, such as amino acid derivatives and dufulin, enhance host resistance by regulating defense-related gene expression and enzymatic activity [20, 21].

Compounds containing pyrimidine and guanidine moieties represent a promising class of antiviral agents with significant potential for managing plant viral diseases. Recent studies have shown that these structures exhibit diverse mechanisms of action, including interference with viral replication, enhancement of host resistance, and disruption of viral protein–nucleic acid interactions [22–24]. Specifically, our previous study demonstrated that a novel pyrimidine morpholine guanidine compound GLY-15 significantly inhibited the infection of tobacco mosaic virus (TMV) [23]. Similarly, guanidine-based compounds, known for their cationic properties, can inhibit viral proliferation by targeting early stages of viral replication, as observed in studies on human and animal viruses [25, 26]. Furthermore, hybrid molecules combining pyrimidine and guanidine functionalities have shown enhanced antiviral activity, suggesting a synergistic effect that could be leveraged for plant virus control [27]. Thus, pyrimidine- and guanidine-based compounds represent promising candidates for the development of novel and sustainable antiviral solutions in agriculture.

Understanding the molecular mechanisms underlying host–virus interactions and the mode of action of antiviral compounds is essential for optimizing their application in disease management. Transcriptomic analysis has proven to be a powerful tool for dissecting the global gene expression changes in plants during viral infection and in response to chemical treatments. By identifying differentially expressed genes (DEGs) and enriched biological pathways, transcriptomics can provide insights into key regulatory networks involved in host resistance and compound-mediated antiviral responses [28–30]. Furthermore, virus-induced gene silencing (VIGS) is a valuable tool for functional validation of candidate genes involved in plant resistance to viruses.

Activity-based protein profiling (ABPP) technology can directly capture and identify drug targets. Compared to traditional transcriptome or proteomic methods, ABPP is particularly suitable for revealing the target proteins of small molecule compounds in complex biological systems, and it has been successfully applied in target discovery of various plant protectants [31]. Research has shown that ABPP has identified β-ketoacyl-ACP-synthase II (FabF) as the first potential target of quinazoline derivatives [32]. Additionally, it has confirmed the translation regulator (CsrA) and virulence regulator (Xoc3530) as effective targets for the plant protectant 1,3,4-oxadiazole thioether A_10_ [33].

In our previous research, a highly active intermediate 1.3-dione was screened by replacing the amino group on the pyrimidine ring with phenylbiguanide, and 25 novel structures of phenylbiguanide compounds were synthesized [34]. The activity assays showed that the new compounds YYH-6, YYH-8, and YYH-9 effectively inhibited the necrotic lesions caused by TMV on *Nicotiana glutinosa* [34]. In this study, we systematically evaluated the antiviral activity of a series of novel phenylbiguanide compounds against TYLCV in *S. lycopersicum* cv. Micro-Tom, and identified YYH-6 with the highest antiviral activity via phenotypic observation and qPCR quantification. By integrating multiple approaches including transcriptomic analysis, real-time quantitative RT-PCR (RT-qPCR), activity-based protein profiling (ABPP), mass spectrometry-based identification, and virus-induced gene silencing (VIGS), we identified *RPP13* and *ALS2* as key defensive genes against TYLCV whose expression is induced by YYH-6, while STI1CP and SHMT were verified as potential target proteins of the compound. The findings of this study provide critical insights into the molecular mechanism of YYH-6, highlighting its innovative potential as an antiviral agent for the sustainable management of TYLCV in tomato production.

## Results

### Y‌YH-6 exhibits potent antiviral activity against TYLCV in tomato plants

**Figure 1:**
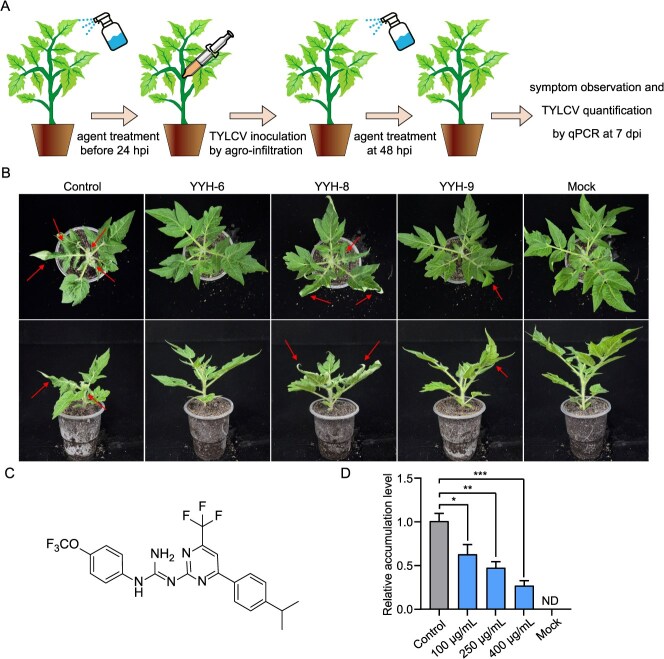
Inhibitory activity of novel phenylpyrimidine guanidine compounds against TYLCV. (A) Screening method for antiviral active compounds. (B) Leaves of TYLCV-inoculated Micro-Tom tomato plants treated with control, 500 μg/ml YYH-6, YYH-8, or YYH-9. (C) Chemical structural formula of YYH-6. (D) qPCR analysis of the accumulation of viral DNA of TYLCV from tomato that was treated with control, 100, 200, and 400 μg/ml YYH-6 at 7 dpi.

To evaluate the anti-TYLCV efficacy of synthetic compounds, we selected 13 compounds from the YYH series with reported antiviral and antibacterial activities for anti-TYLCV activity assays, and treated *S. lycopersicum* cv. Micro-Tom with candidate agents following a staged application protocol (Fig. 1A): preinoculation treatment (24 h before TYLCV agro-infiltration), viral inoculation, and postinoculation treatment (48 h postinoculation), followed by symptom observation and qPCR-based viral quantification at 7 days postinoculation (dpi). The results demonstrated that the YYH series compounds exhibited varying levels of anti-TYLCV activity (Fig. S1). Compounds including 500 μg/ml of YYH-6, YYH-8, and YYH-9 with distinct activity profiles were selected as representative examples for subsequent analysis (Fig. 1B). From a phenotypic perspective, the control tomato plants exhibited typical disease symptoms like leaf curling and plant dwarfism, while the plants treated with YYH-6 did not show any observable disease symptoms (Fig. 1B). Plants treated with YYH-8 and YYH-9 showed more severe symptoms than those treated with YYH-6 and milder symptoms than the control (Fig. 1B). Analysis of viral accumulation by qPCR further confirmed that YYH-6 has the highest anti-TYLCV activity among the tested compounds, reducing TYLCV accumulation in the upper systemic leaves by 83.26% compared with the control group (Fig. S1). Then, different concentrations of YYH-6 were applied to further determine the effective anti-TYLCV activity (Fig. 1C). The qPCR results showed that TYLCV infection was progressively inhibited with increased concentration of YYH-6 from 100 to 400 μg/ml, with the inhibition rate ranging from 37.3% to 77.2% (Fig. 1D).

### RNA-seq analysis uncovers critical genes and pathways involved in YYH-6-induced antiviral responses

**Figure 2:**
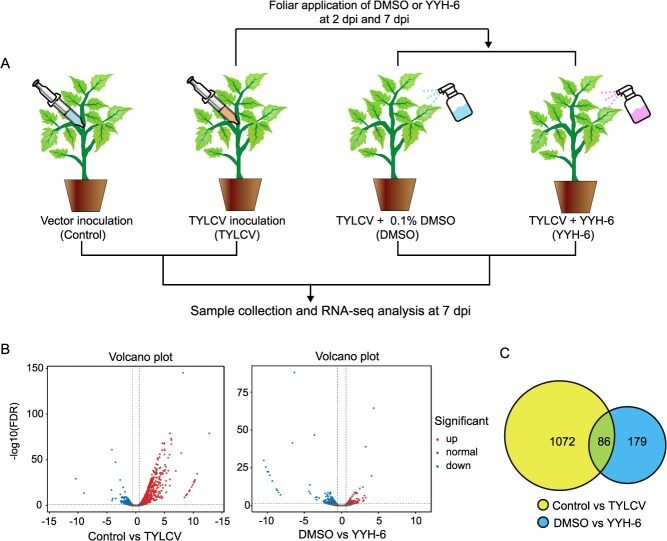
Transcriptomic analysis of Micro-Tom tomato plants in response to YYH-6 treatment. (A) Schematic of the experimental design for Micro-Tom tomato transcriptomic analysis. (B) Volcano plot of DEGs: control vs TYLCV, DMSO vs YYH-6. (C) Venn diagram of DEGs between TYLCV-infected and TYLCV-infected + YYH-6-treated tomato plants.

To explore the molecular mechanism by which YYH-6 regulates tomato resistance to TYLCV, four treatment groups were set up for RNA sequencing (RNA-seq): control (healthy Micro-Tom tomato plants inoculated with buffer), TYLCV (plants inoculated only with TYLCV), DMSO (plants treated with 0.1% DMSO after TYLCV inoculation), and YYH-6 (plants treated with YYH-6 after TYLCV inoculation). The precise experimental procedure is shown in Fig. 2A. DEGs were screened by pairwise comparison with the criteria of |log₂ fold change| > 1 and adjusted *P* value <0.05.

Volcano plot analysis showed that compared with the control group, a total of 1158 DEGs were identified in the TYLCV group, including 871 significantly upregulated genes and 287 significantly downregulated genes (Fig. 2B, Table S3). In the comparison between the DMSO group and the YYH-6 group, a total of 265 DEGs were identified, including 107 upregulated genes and 158 downregulated genes (Fig. 2B, Table S4). This suggested that YYH-6 treatment could regulate the expression changes of some genes caused by TYLCV infection. In addition, Venn diagram analysis further clarified YYH-6-regulated core genes. Among 1158 DEGs induced by TYLCV infection, 86 (7%) exhibited significant expression changes upon YYH-6 treatment (Fig. 2C, Table S5). These YYH-6-responsive DEGs are potentially key to YYH-6-mediated antiviral responses.

**Figure 3:**
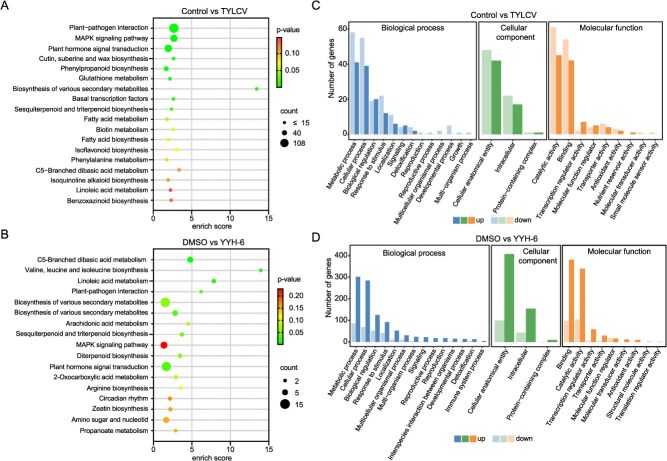
KEGG analysis of DEGs: (A) control vs TYLCV and (B) DMSO vs YYH-6. GO analysis of DEGs: (C) control vs TYLCV and (D) DMSO vs YYH-6. Each spot represents a KEGG pathway. The ordinate represents the pathway name and the abscissa represents the enrichment factor, which represents the ratio of the proportion of genes annotated to a pathway to all genes annotated to that pathway.

KEGG pathway enrichment analysis was performed on the DEGs from the two comparison groups (control vs TYLCV and DMSO vs YYH-6) (Fig. 3A and B). The results showed that the DEGs in the control vs TYLCV group were significantly enriched in pathways such as ‘plant–pathogen interaction’, ‘MAPK signaling pathway’, ‘plant hormone signal transduction’, and ‘phenylpropanoid biosynthesis’ (*P* < 0.05). Among these, the ‘plant–pathogen interaction’ pathway had the largest number of enriched genes, indicating that TYLCV infection mainly interfered with the immune defense-related pathways of tomatoes.

In the DMSO vs YYH-6 group, the DEGs were preferentially enriched in pathways including ‘C5-branched dibasic acid metabolism’, ‘linoleic acid metabolism’, ‘sesquiterpenoid and triterpenoid biosynthesis’, and ‘plant–pathogen interaction’ (Fig. 3B). Notably, YYH-6 treatment significantly enhanced the expression of key genes (e.g. PR genes, disease resistance protein genes) in the ‘plant–pathogen interaction’ pathway and simultaneously activated fatty acid metabolism and secondary metabolism-related pathways. This suggested that YYH-6 might enhance tomato antiviral ability by regulating immune pathways and metabolic reprogramming.

GO functional enrichment analysis classified DEGs into three major categories: ‘biological process’, ‘cellular component’, and ‘molecular function’ (Fig. 3C and D). In the control vs TYLCV group, under the ‘biological process’ category, DEGs were mainly enriched in ‘defense response’ and ‘response to stimulus’; under the ‘molecular function’ category, genes related to ‘catalytic activity’ and ‘binding’ accounted for the highest proportion, and most of these genes were downregulated (Fig. 3C). After YYH-6 treatment, genes related to ‘metabolic process’ and ‘immune response’ in the ‘biological process’ category were significantly upregulated, and the enrichment degree of genes related to ‘antioxidant activity’ and ‘transcription regulator activity’ in the ‘molecular function’ category was significantly increased (Fig. 3D).

### RT-qPCR validation and functional analysis of key genes in YYH-6-mediated tomato resistance

**Figure 4:**
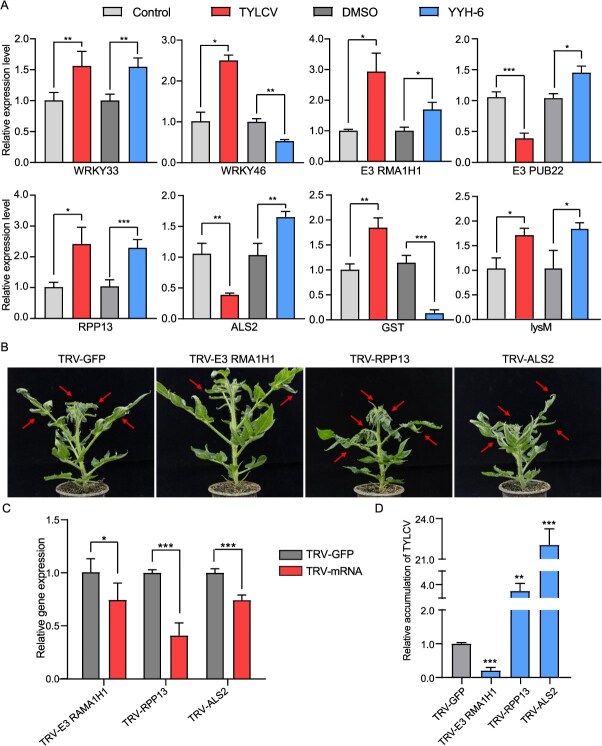
Validation of RNA-seq results and functional characterization of candidate genes in Micro-Tom tomato response to TYLCV infection. (A) RT-qPCR verification of the regulation of 8 DEGs. (B) Symptoms of tomato infected with TYLCV after gene silencing. RT-qPCR analysis of (C) gene silencing efficiency and (D) TYLCV expression in tomato.

We selected eight DEGs related to transcription factors, disease resistance, ubiquitination, and defense enzymes from the RNA-seq dataset, and validated their expression via RT-qPCR. The results indicated that the expression trends of all candidate genes were consistent with the RNA-seq data, confirming the accuracy and reliability of the transcriptome sequencing results. Specifically, compared with the control group, TYLCV infection significantly downregulated the expression of genes such as *E3 ubiquitin-protein ligase PUB22-like* (*E3 PUB22*, LOC101267151) and *acetolactate synthase 2* (*ALS2*, LOC101254112), while upregulating the expression of *WRKY transcription factor 33* (*WRKY33*, LOC101268787), *WRKY transcription factor 46* (*WRKY46*, LOC101268780), *E3 ubiquitin-protein ligase RMA1H1* (*E3 RMA1H1*, LOC101262152), *disease resistance RPP13-like protein* (*RPP13*, LOC101267186), *glutathione S transferase* (*GST*, LOC101244300), and *lysM domain receptor-like kinase* (*lysM*, LOC101261978) (Fig. 4A). After YYH-6 treatment, the expression levels of *WRKY33*, *E3 RMA1H1*, *E3-PUB22*, *RPP13*, *ALS2*, and *lysM* were significantly upregulated, and the expression of *WRKY46* and *GST* was significantly downregulated (Fig. 4A). Collectively, these results suggest that YYH-6 can regulate the expression of important resistance or defense-related host genes during TYLCV infection.

To further validate the roles of several potentially crucial DEGs regulated by YYH-6 in tomato-TYLCV interaction, we employed tobacco rattle virus (TRV)-mediated virus-induced gene silencing (VIGS) to downregulate *E3 RMA1H1*, *RPP13*, and *ALS2* in tomato plants, with TRV-GFP serving as the negative control. RT-qPCR analysis confirmed that the expression levels of *E3 RMA1H1*, *RPP13*, and *ALS2* were reduced by 55%–72% in the corresponding VIGS lines compared with those in the TRV-GFP (control), indicating efficient gene silencing (Fig. 4C). Compared with the control plants, *RPP13*- and *ALS2*-silenced plants exhibited significantly exacerbated severe stunting and leaf curl symptoms following TYLCV inoculation, whereas *E3 RMA1H1*-silenced plants displayed significantly milder viral symptoms (Fig. 4B). Consistent with the phenotypic observations, the results of qPCR revealed that silencing of *RPP13* and *ALS2* resulted in a 4.2-fold and 22.4-fold increase in TYLCV accumulation, respectively, whereas the accumulation levels of TYLCV in *E3 RMA1H1*-silenced plants were significantly lower than those in the TRV-GFP control group (Fig. 4D). These findings suggest that *RPP13* and *ALS2* are critical YYH-6-induced genes that positively regulate tomato resistance to TYLCV. Additionally, several crucial DEGs, such as *WRKY33*, *E3 PUB22*, *GST*, and *lysM*, were also induced by YYH-6. The function of these genes in resistance to TYLCV needs to be further investigated in the following study.

### Synthesis of biotin-labeled probe and affinity identification of the potential target proteins of YYH-6

**Figure 5:**
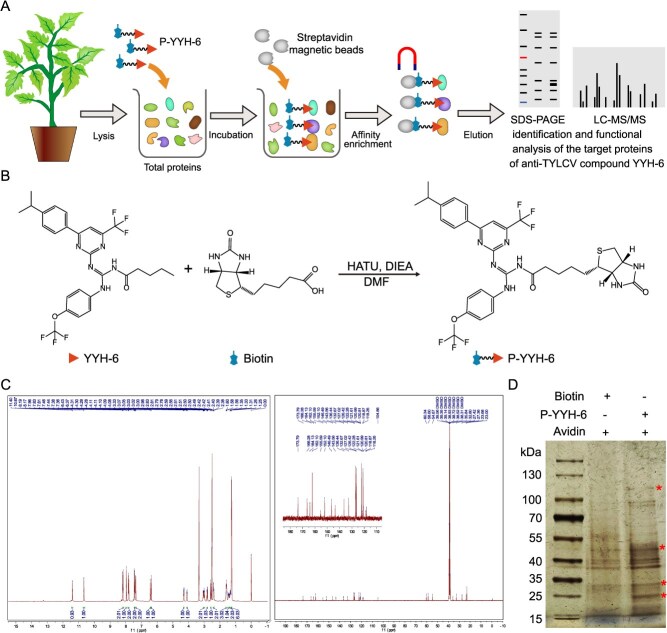
Identification of potential target proteins of YYH-6. (A) Schematic experimental procedure for affinity enrichment and identification of YYH-6 target proteins: tomato total proteins were incubated with P-YYH-6, followed by streptavidin magnetic bead-based affinity enrichment, elution, SDS-PAGE separation, and LC–MS/MS identification. (B) Synthesis of P-YYH-6. (C) ^1^H NMR and ^13^C NMR of compound P-YYH-6. (D) SDS-PAGE verification of affinity enrichment: specific protein bands (marked with *), enriched only in the P-YYH-6 treatment group (right lane), indicate potential target proteins of YYH-6 (biotin group: negative control; avidin: streptavidin magnetic beads).

To elucidate the molecular mechanism underlying the activity of compound YYH-6 against TYLCV, this study designed and synthesized a biotin-labeled YYH-6 probe (P-YYH-6) and combined affinity enrichment with mass spectrometry to identify its target proteins. YYH-6 and biotin were used as substrates to synthesize P-YYH-6 via an amidation reaction in DMF catalyzed by HATU and DIEA (Fig. 5B). Subsequently, ^1^H NMR and ^13^C NMR were used to characterize its chemical structure, and the presence of characteristic hydrogen and carbon signals in the spectra confirmed the structural accuracy of P-YYH-6, providing a reliable probe molecule for subsequent affinity enrichment experiments (Fig. 5C). Moreover, anti-TYLCV efficacy of P-YYH-6 was analyzed using Micro-Tom plants. P-YYH-6 exhibited antiviral activity comparable to that of YYH-6, achieving a 51.7% inhibition rate against TMV at a concentration of 250 μg/ml (Fig.   S2).

Next, an affinity enrichment protocol was established in this study for the identification of YYH-6 target proteins (Fig. 5A). Total proteins were first isolated from TYLCV-infected Micro-Tom tomato leaves by lysis and incubated with P-YYH-6 to achieve specific binding between the probe and target proteins. Then, streptavidin magnetic beads were added to capture the target protein complexes through the high-affinity biotin–avidin interaction for affinity enrichment. After elution, the target proteins were identified by SDS-PAGE separation and LC–MS/MS analysis. To verify the specific binding of P-YYH-6 to target proteins, silver staining analysis (Fig. 5D) was performed, and the results showed that specific protein bands (marked with asterisks) appeared only in the P-YYH-6 treatment group, while no obvious bands were observed in the biotin control group, confirming that P-YYH-6 can specifically bind to its target proteins and verifying the effectiveness of the affinity probe.

Through LC–MS/MS analysis of the affinity-enriched proteins, we identified multiple candidate target proteins, including families such as thioredoxin domain-containing protein (TXNDC), STI1 domain-containing protein (STI1CP), serine hydroxymethyltransferase (SHMT), and peptidase S9 prolyl oligopeptidase catalytic domain-containing protein (S9POPcatCP) (Tables 1 and S6). These proteins are mainly involved in various biological processes such as redox regulation, protein folding, amino acid metabolism, and enzymatic catalysis (Table S6).

**Table 1:** YYH-6-binding proteins identified by the ABPP technique.

**Gene IDs**	**Protein names**	**Protein IDs**	**Mol. weight**
LOC101251929	Thioredoxin domain-containing protein (TXNDC)	A0A3Q7EP13	117.06 kDa
LOC101268416	STI1 domain-containing protein (STI1CP)	A0A3Q7HV74	45.60 kDa
LOC101263138	Serine hydroxymethyltransferase (SHMT)	A0A3Q7HN29	56.99 kDa
LOC101255389	Peptidase S9 prolyl oligopeptidase catalytic domain-containing protein (S9POPcatCP)	A0A3Q7G4G6	80.58 kDa

### Characterization of YYH-6 binding proteins and their impacts on TYLCV infection

**Figure 6:**
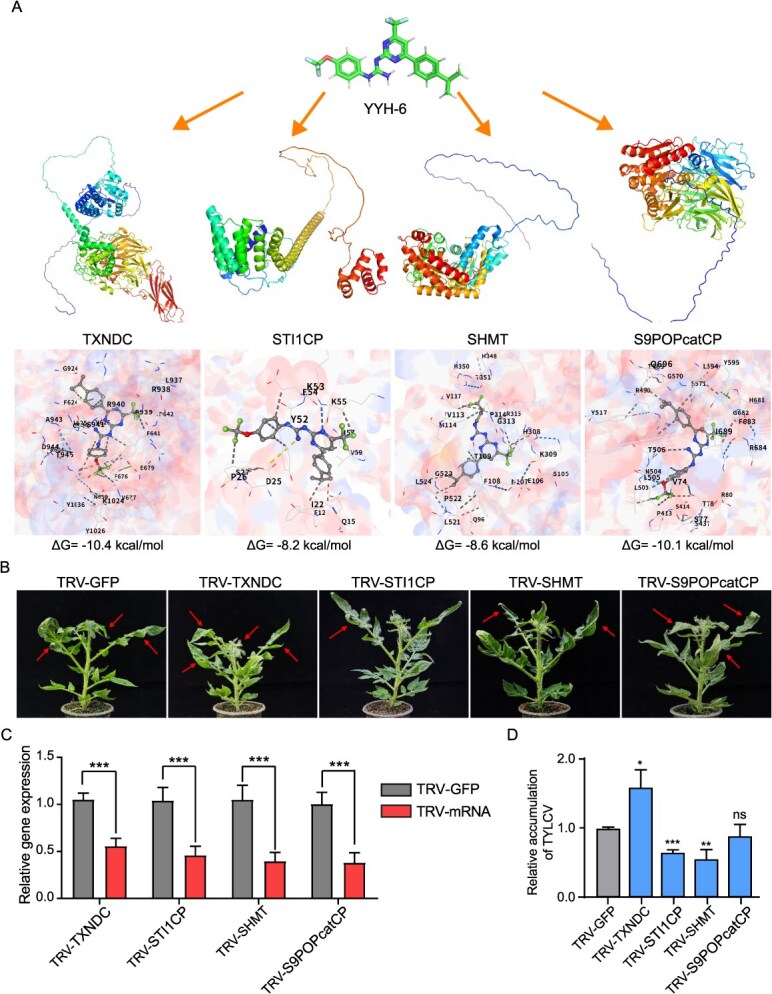
Molecular docking of YYH-6 with target proteins and functional analysis of candidate genes in tomato response to TYLCV. (A) Molecular docking results of YYH-6 with target proteins, including TXNDC, STI1CP, SHMT, and S9POPcatCP. (B) Symptoms of tomato infected with TYLCV after gene silencing. RT-qPCR analysis of (C) gene silencing efficiency and (D) TYLCV expression in tomato.

To investigate the potential interaction between potential target proteins and YYH-6, molecular docking assays were performed (Fig. 6A). The results showed that TXNDC, STI1CP, SHMT, and S9POPcatCP exhibited strong binding affinities to the target YYH-6 (ΔG values of −10.4, −8.2, −8.6, and −10.1 kcal/mol, respectively) (Fig. 6A). The predicted critical amino acid residues mediating the interaction between YYH-6 and its target protein were presented in Table S7. Phenotypic observation revealed that the gene-silenced tomato plants exhibit distinct responses to TYLCV infection. Compared with the TRV-GFP control, TRV-TXNDC plants showed obvious leaf curl symptoms, whereas TRV-STI1CP, TRV-SHMT plants displayed relatively milder phenotypes (Fig. 6B). RT-qPCR analysis validated that the expression levels of *TXNDC*, *STI1CP*, *SHMT*, and *S9POPCATCP* in TRV-mRNA plants were significantly reduced compared with the TRV-GFP control, confirming substantial silencing efficiency for each genes (Fig. 6C). Consistent with the phenotypic observations, the accumulation levels of TYLCV genome DNA in the *TXNDC*-silenced plants were significantly higher than those in the TRV-GFP control group, whereas *STI1CP* and *SHMT* silencing reduced virus accumulation by 46% and 52%, respectively (Fig. 6D). These results indicated that STI1CP and SHMT function as susceptibility host genes (beneficial for TYLCV infection) potentially targeted by YYH-6.

## Discussion

The persistent threat posed by TYLCV to global tomato production necessitates the development of novel and sustainable antiviral strategies. Currently, insecticide-based control targeting viral vectors represents the mainstream approach, yet its efficacy is compromised by the dual challenges of insecticide resistance and adverse environmental impacts [35]. Beyond conventional insecticides, a variety of synthetic and natural compounds have been explored for their direct or indirect antiviral activities against plant viruses. Dufulin, a representative plant immune inducer, applied as a 20% formulation, achieves an average field control effect of 68.29% against TYLCV, mainly by activating the SA signaling pathway and enhancing defense enzyme activities to trigger host resistance of tomato plants [20, 36]. In addition, amino acid derivatives and other natural or synthetic compounds have been reported to inhibit TYLCV replication and assembly or trigger host immune responses through diverse antiviral mechanisms [17, 37]. To date, effective compounds for effectively controlling TYLCV remain limited. In this study, we systematically characterized the key resistance genes induced by YYH-6 and identified its potential targets during TYLCV infection.

Transcriptomic analysis provided the first layer of insight into YYH-6’s mode of action. TYLCV infection caused a widespread suppression of the host transcriptome, particularly affecting genes involved in ‘plant–pathogen interaction’, ‘MAPK signaling’, and ‘Plant hormone signal transduction’. This large-scale downregulation likely facilitates viral establishment and systemic infection. Strikingly, YYH-6 treatment regulated the expression of a significant subset of these virus-altered genes. The upregulation of defense-related genes such as *WRKY46*, *RPP13*, and *GST* upon YYH-6 treatment, coupled with the enrichment of DEGs in immune and metabolic pathways, suggests that YYH-6 acts as an immune response inducing agent. This is consistent with the mode of action reported for other resistance inducers like dufulin [20] and certain amino acid derivatives [21], but the specific gene set modulated by YYH-6 appears distinct.

Plant disease resistance is a critical trait for global food security, with sophisticated defense mechanisms evolved by plants to counter pathogen attacks [38]. Among these mechanisms, Nucleotide-Binding Site-Leucine-Rich Repeat (NBS-LRR) proteins play a crucial role in initiating immune responses by recognizing pathogen-derived signals [39, 40]. Leucine-rich repeat (LRR) proteins can be categorized into several groups, such as LRR receptor-like kinases (LRR-RLKs), LRR receptor-like proteins (LRR-RLPs), and NBS-LRRs [40]. RPP13-like proteins, belonging to the NBS-LRR class, are particularly important for recognizing specific pathogen effectors and activating plant immune responses. The RPP13 gene family is well characterized for its pivotal role in plant disease resistance responses [41]. For instance, comprehensive identification and analysis of 28 RPP13 gene family members in potato (*S. tuberosum* L.) have been conducted and demonstrated their functions in disease resistance [41]. Similarly, a novel CC-NBS-LRR gene, NtRPP13, from tobacco (*N. tabacum*), has been shown to positively regulate hypersensitive response and phytohormone-related defense genes, conferring enhanced resistance to bacterial wilt disease caused by *Ralstonia solanacearum* [42]. In this study, transcriptome sequencing showed that *RPP13* is significantly upregulated in TYLCV-infected plants. TRV-VIGS-mediated *RPP13* silencing results reveals that *RPP13* positively regulates plant resistance to TYLCV. Acetolactate synthase (ALS), also known as acetohydroxyacid synthase (AHAS), is a crucial enzyme in the biosynthesis pathway of branched-chain amino acids (BCAAs), including valine, leucine, and isoleucine, in both plants and microorganisms [43, 44]. In plants, ALS plays a pivotal role in mediating BCAA biosynthesis within chloroplasts, a process indispensable for normal plant growth and development [45, 46]. ALS also serves as a primary molecular target of multiple commercial herbicides [47, 48], and accumulating evidence further links this enzyme to the modulation of plant responses to biotic stress. In tomato, downregulation of acetolactate synthase reduces Ol-1-mediated resistance to powdery mildew, suggesting a potential role for ALS in plant immune responses or disease resistance mechanisms [49]. In this study, the transcriptome profiling data reveal significant upregulation of *ALS2* in YYH-6-treated plants, and TRV-VIGS-mediated silencing of *ALS2* resulted in elevated TYLCV accumulation. These results collectively validate *RPP13* and *ALS2* as critical genes whose expression is regulated by YYH-6 and involved in resistance to TYLCV.

ABPP technology successfully identified multiple potential target proteins of YYH-6. Among these, thioredoxin domain-containing proteins (e.g. TXNDC) serve as key regulators of plant redox homeostasis [50]. Dysfunction of TXNDC leads to imbalanced accumulation of reactive oxygen species, thereby impairing the host resistance response [51]. Serine hydroxymethyltransferase (e.g. SHMT), a core enzyme in the one-carbon metabolism pathway, is not only involved in amino acid synthesis but also plays a crucial role in plant responses to environmental stresses [52, 53]. In addition, STI1 domain-containing proteins (e.g. STI1CP) participate in protein folding and chaperone functions [54]. The functional diversity of these potential target proteins indicates that YYH-6 may regulate the host’s metabolic and defense networks through a multitarget synergistic mechanism. Specifically, silencing of *TXNDC* markedly increases viral accumulation, while *STI1CP* or *SHMT* silencing suppresses viral accumulation of TYLCV. This observation not only highlights the unique roles of individual targets in viral infection but also emphasizes the complexity inherent in YYH-6-mediated multitarget regulation. SHMT is a hub enzyme linking carbon metabolism and amino acid biosynthesis, both of which are essential for viral genome replication and capsid protein synthesis. YYH-6 may target SHMT to restrict the supply of metabolic intermediates required by TYLCV, thus suppressing viral accumulation. As for STI1CP, its chaperone function is likely involved in the folding and assembly of viral proteins or host defense-related proteins; YYH-6-mediated inhibition of STI1CP may impair viral protein maturation, leading to reduced viral loads.

In this study, no direct interaction between YYH-6 and viral proteins encoded by TYLCV were identified in the results of ABPP. However, we do not exclude the possibility of weak binding affinity of YYH-6 with TYLCV genomes or proteins, which should be investigated in our following study. Furthermore, interaction between the potential targets of YYH-6 and TYLCV proteins also requires in-depth analysis in subsequent experiments to further validate the antiviral mechanisms of the compound.

## Conclusion

**Figure 7:**
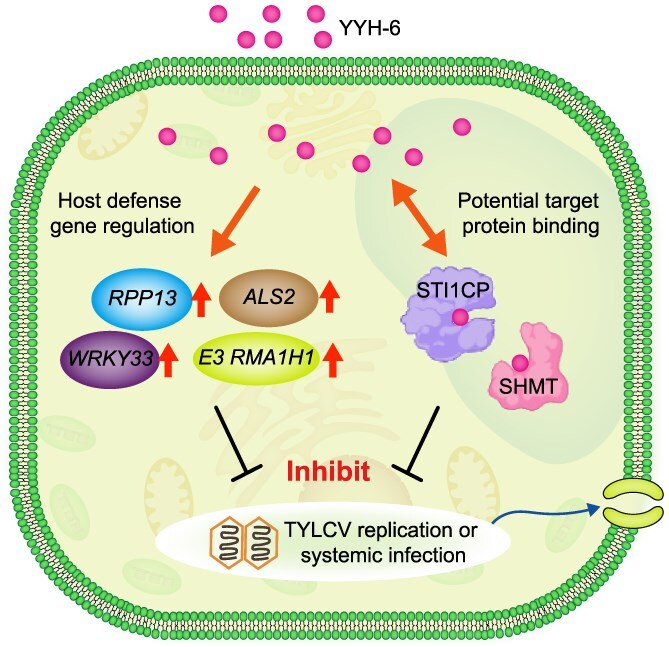
Model for host defense gene regulation and potential target protein binding mediating the inhibition of TYLCV replication or systemic infection by YYH-6. YYH-6 modulates host defense genes, including *RPP13*, *WRKY33*, *ALS2*, and *E3 RMA1H1*, and has potential binding with target proteins such as STI1CP and SHMT, which collectively exert an inhibitory effect on the replication or systemic infection of TYLCV.

In conclusion, our integrated study reveals YYH-6 as a promising anti-TYLCV candidate that operates through a multifaceted mechanism (Fig. 7). Mechanistically, transcriptomic analysis showed YYH-6 modulates TYLCV-disrupted host transcription, enriching defense pathways including ‘plant–pathogen interaction’ and secondary metabolism, while TRV-VIGS validation indicates that *RPP13* and *ALS2* function as resistance genes against TYLCV induced by YYH-6. Additionally, analysis of ABPP, LC–MS/MS, molecular docking, and functional analysis by VIGS revealed STI1CP and SHMT as potential targets of YYH-6. Collectively, our findings reveal the multipathway regulatory mechanism of YYH-6 in antiviral responses against TYLCV, providing a theoretical basis for the development of novel antiviral strategies and the application of this novel active compound in viral disease management.

## Materials and methods

### Plant materials and growth conditions

Seeds of tomato (*S. lycopersicum* L. cv. Micro-Tom) were surface sterilized via 55°C warm water soaking for 15 min, then germinated in the dark at 30°C until radicle emergence. Following transplantation, the seedlings were under controlled conditions of 25°C, a 16/8-hour light/dark cycle, and 60%–70% relative humidity. Four-week-old tomato seedlings with three to four fully expanded true leaves were selected for all experiments.

### Virus strain and chemical reagent

TYLCV Beijing isolate (TYLCV-BJ, PMID: 34257307) infectious clone was provided by Prof. Xueping Zhou (Chinese Academy of Agricultural Sciences). *Agrobacterium tumefaciens* strain GV3101 harboring the TYLCV-BJ clone was used for inoculation. A series of novel phenylpyrimidine guanidine compounds (100 mg/ml) were prepared by dissolving them in dimethyl sulfoxide (DMSO, Sigma-Aldrich, USA), and these were diluted to the working concentration (500 μg/ml) with sterile distilled water; the final concentration of DMSO in the working solution was 0.1% (v/v). The control group was treated with 0.1% DMSO aqueous solution.

### TYLCV inoculation and compound treatment

Tomato seedlings were first foliar sprayed with 500 μg/ml compounds 24 h prior to TYLCV inoculation, which were expressed through agrobacterium stem injection. At 48 h postinoculation (48 hpi), a second spray of compounds were applied. The control group was treated with 0.1% DMSO aqueous solution. Phenotypic symptoms were observed and photographed at 7 dpi. Finally, the upper leaves were collected for DNA extraction and qPCR analysis for virus quantification.

### Transcriptomics analysis

For transcriptome analysis, 4-week-old tomato seedlings were inoculated with the TYLCV-BJ isolate, while control plants were mock inoculated with an empty agrobacterium vector. Systemic leaves were collected from both groups at 7 days postinoculation (dpi) for subsequent RNA sequencing. In a separate set of experiments, 4-week-old tomato seedlings were inoculated with TYLCV-BJ. At 2 dpi, plants were divided into two groups and foliar sprayed with either a 500 μg/ml YYH-6 or 0.1% DMSO, respectively. A second round of spraying was performed at 7 dpi. Systemic leaves were sampled for RNA sequencing at 6 h after the second spraying. This time point was selected according to the early infection characteristics of TYLCV and our preliminary experimental data, which is sufficient to detect the early transcriptional responses of tomato plants to YYH-6 treatment against TYLCV infection.

Total RNA was extracted using Magzol Reagent. RNA quality was verified by 1% agarose gel electrophoresis, NanoDrop (A260/A280 1.8–2.0, A260/A230 > 1.8), and Agilent 2100 Bioanalyzer (RIN ≥8.0). Libraries were constructed with Illumina TruSeq Kit, and 150-bp paired-end sequencing was performed on NovaSeq 6000, generating ~6 Gb raw data/sample. The raw reads generated by Illumina sequencing were submitted to the Sequence Read Archive database at NCBI (SRA, http://www.ncbi.nlm.nih.gov/Traces/sra) with the SRA BioProject accession number PRJNA1271683. Clean reads were aligned to tomato genome (SL4.0) using hierarchical indexing for spliced alignment of transcripts 2 with StringTie assembly and fragments per kilobase of transcript per million mapped fragments (FPKM) quantification via featureCounts. DEGs were identified by DESeq2 (|log₂FC| > 1, adjusted *P* < 0.05), visualized via volcano plots/Venn diagrams. GO enrichment (ClusterProfiler) and KEGG pathway analysis (KOBAS) were performed, with significant terms filtered by adjusted *P* < 0.05 and visualized as bar/dot plots.

### Design, synthesis, and characterization of a biotin-labeled YYH-6 probe

Based on the structural features of YYH-6 and biotin, combined with software simulations, the biotin-YYH-6 probe was designed as a molecular tool for affinity labeling studies. Briefly, a mixture of YYH-6 (1.0 mmol), biotin (1.0 mmol), HATU (1.5 mmol), and DIEA (1.5 mmol) in DMF (2 ml) was added to a 50-ml round-bottom flask. The crude product was obtained by purification of the reaction mixture via preparative liquid chromatography (C18 column) using a gradient of ACN/H₂O (0.15% NH₃·H₂O, v/v = 2/1) as the eluent. This crude product was then dissolved in a mixed solvent of ACN (5 ml) and H₂O (5 ml) at 60°C. Upon slow cooling to room temperature, the target product gradually precipitated and was isolated by filtration to afford 0.1 g of a white solid, corresponding to the desired biotin-YYH-6 probe. The structure of the synthesized biotin-YYH-6 probe was unequivocally confirmed by ^1^H NMR and ^13^C NMR spectroscopy.

### Isolation of probe-binding proteins by streptavidin magnetic beads

Total proteins were extracted from the upper leaves of TYLCV-BJ-inoculated tomato plants (14 days postinoculation) by grinding the tissue in liquid nitrogen, followed by homogenization in protein extraction buffer and centrifugation. The resultant supernatant was incubated with either the biotin-YYH-6 probe or biotin alone (50 μM each) at room temperature for 1 h. Meanwhile, streptavidin magnetic beads were washed and equilibrated with TBS. The protein–probe mixtures were then incubated with the prepared beads for 1 h to capture the binding complexes. After extensive washing, the bound proteins were eluted by boiling in SDS-PAGE sample buffer for subsequent gel electrophoresis and mass spectrometric analysis.

### SDS-PAGE and LC–MS/MS analysis

The eluted proteins were denatured with loading buffer, separated by SDS-PAGE under constant voltage (150 V, 50 min), and visualized by silver staining, which involved sequential fixation, sensitization, silver impregnation, and development steps. For protein identification, the excised gel bands were subjected to in-gel tryptic digestion (37°C, 16–18 h), and the resulting peptides were analyzed by LC–MS/MS on a Q Exactive HF-X mass spectrometer coupled to a nanoflow HPLC system with a C18 column, using a 60-min gradient of acetonitrile in 0.1% formic acid. Data were processed using MaxQuant software (v1.5.5.1) against a combined tomato and TYLCV database.

### TRV-based virus induced gene silencing

Target gene fragments (200–300 bp) were cloned into the TRV2 vector using EcoRI/BamHI restriction sites, and recombinant vectors (pTRV2-WRKY46, pTRV2-RPP13, etc.) were transformed into *A. tumefaciens* strain GV3101; TRV1 and recombinant TRV2 (1:1 v/v, OD_600_ = 1.0) were then co-infiltrated into two to three true leaf-stage Micro-Tom seedlings, with TRV-GFP used as a control, followed by RNA extraction at 14 dpi to detect silencing efficiency via RT-qPCR. After gene silencing validation, TYLCV-BJ was inoculated and tomato upper leaves were collected 7 days later for DNA extraction and qPCR detection of virus accumulation. The primers used to construct the vector were listed in Table S1.

### DNA extraction and viral quantification via qPCR

Total DNA was extracted using the CTAB method from TYLCV-inoculated systemic leaves of *S. lycopersicum* cv. Micro-Tom treated with a serial of phenylpyrimidine guanidine compounds or control. Viral accumulation was quantified via real-time PCR (qPCR) with TYLCV CP gene-specific primers (F: 5′-CCCTCAAAGCTCTATGGCAATCGG-3′, R: 5′-CAGTGACGTCTGTGGAACCCTC-3′) and tomato actin as the internal reference gene (F: 5′-AGAGCTATGAGCTCCCAGATGG-3′, R: 5′-TTAATCTTCATGCTGCTAGGAGC-3′). Reactions were performed using ChamQ Universal SYBR qPCR Master Mix (Vazyme) on an Applied Biosystems StepOne Plus system, and data were analyzed via the 2^−ΔΔCt^ method.

### Real-time quantitative RT-PCR

Total RNA was extracted from upper leaves using the Eastep® Super Total RNA Extraction Kit (Promega, Shanghai, China). Thereafter, cDNA was synthesized using HiScript III RT SuperMix (+gDNA wiper) (Vazyme). RT-qPCR analysis was performed on a StepOne Plus real-time PCR system (Thermo Fisher Scientific, Waltham, USA). The expression levels of eight DEGs and the silencing efficiency of seven genes were validated by RT-qPCR with gene-specific primers (Table S2). The fold changes in gene expression were calculated using the 2^−ΔΔCt^ method.

### Molecular docking

The three-dimensional structure of the compound YYH-6 was constructed with the ChemOffice 19.0.0.22 software as the ligand molecule, while the crystal structures of the A0A3Q7EP13 (TXNDC), A0A3Q7HV74 (STI1CP), A0A3Q7HN29 (SHMT), and A0A3Q7G4G6 (S9POPcatCP) were obtained from UniProt database as the receptor molecule. Docking of ligand molecules and receptor molecules was performed using molecular docking software AutoDock 4.2.6. Finally, PyMOL 2.2.0 software was used to analyze the docking results.

### Statistical analysis

The data were presented as mean ± standard error of three biological replicates and analyzed by Student’s *t* test procedure using SPSS software 17.0 (SPSS, Chicago) with statistical significance (*P < 0.05, **P < 0.01, ***P < 0.001).

## Acknowledgments

This work was supported by the Foundation of Liaoning Provincial Department of Education (JYTYB2024001), Research and Management of Major Non-Infectious Leaf Spot Diseases (SCYC202513), and Green Prevention and Control Technologies for Insect-transmitted Viral Diseases in Sichuan Province (SCYC202605), Enterprise Doctoral Innovation and Entrepreneurship Program of Yingkou City (YKSCJH2024-020). TYLCV infectious clone was kindly provided by Prof. Xueping Zhou from Chinese Academy of Agricultural Sciences, China. The pTRV2 plasmid was kindly provided by Prof. Yule Liu from Tsinghua University, China.

## Supplementary Material

Web_Material_uhag120

## Data Availability

The data supporting the findings of this work are available within the paper and its supplementary information files.
