# Supplementary material for: A novel pyrimidine–guanidine derivative YYH-6 confers tomato resistance to TYLCV via multitarget regulation of host defense genes and proteins
Source: Hortic Res. 2026 Apr 6;13(8):uhag120. doi: 10.1093/hr/uhag120 (PMC13395516; doi:10.1093/hr/uhag120)
Supplement: Web_Material_uhag120 [file web_material_uhag120.zip › Supporting Data-HR-2026-102.docx]

Supporting Information

**A Novel Pyrimidine-Guanidine Derivative YYH-6 Confers Tomato Resistance to TYLCV via Multi-Target Regulation of Host Defense Genes and Proteins**

Miao Yu^1, #^, Kexin Liu^2, #^, Dongyang Liu^3^, Ting Liu^3^, Lianqiang Jiang^3^, Yuhang Yang^1^, Jing Shu^1^, Zhiping Wang^1^, Zihao Xia^1^, Mengnan An^1, *^, Xinghai Li^1, *^, Yuanhua Wu^1, *^


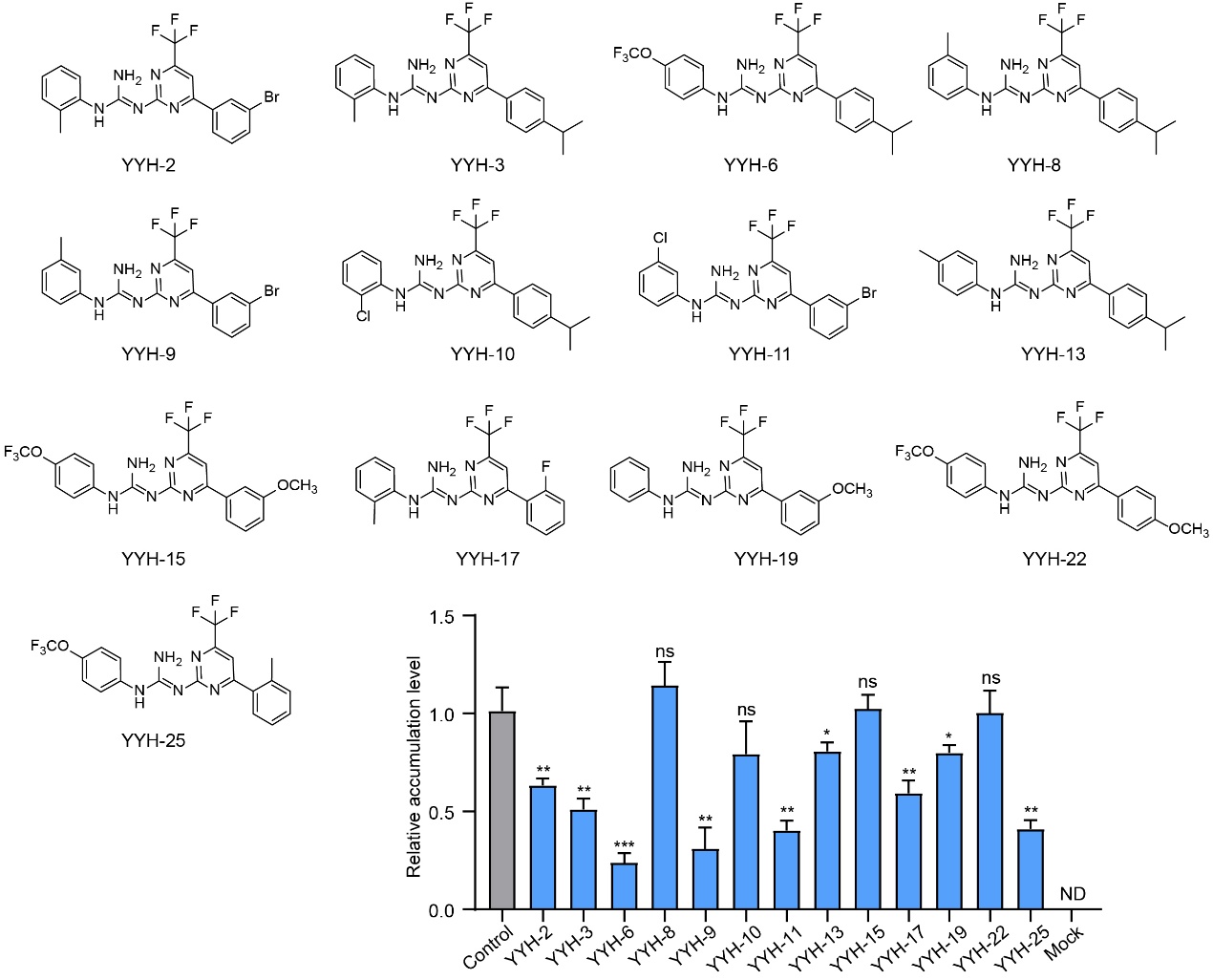


**Figure S1.** Chemical structure of the test compounds and their anti-TYLCV activity in Micro-Tom tomato plants.

**Fig. S2** Anti-TYLCV activity testing of P-GLY-15. (A) Leaves of tomato inoculated with TYLCV by treated by control or 250 μg/mL YYH-6 and P-YYH-6. (D) qPCR analysis of the accumulation of viral DNA of TYLCV from tomato that was treated with control, 250 μg/mL YYH-6 and P-YYH-6 at 7 dpi.
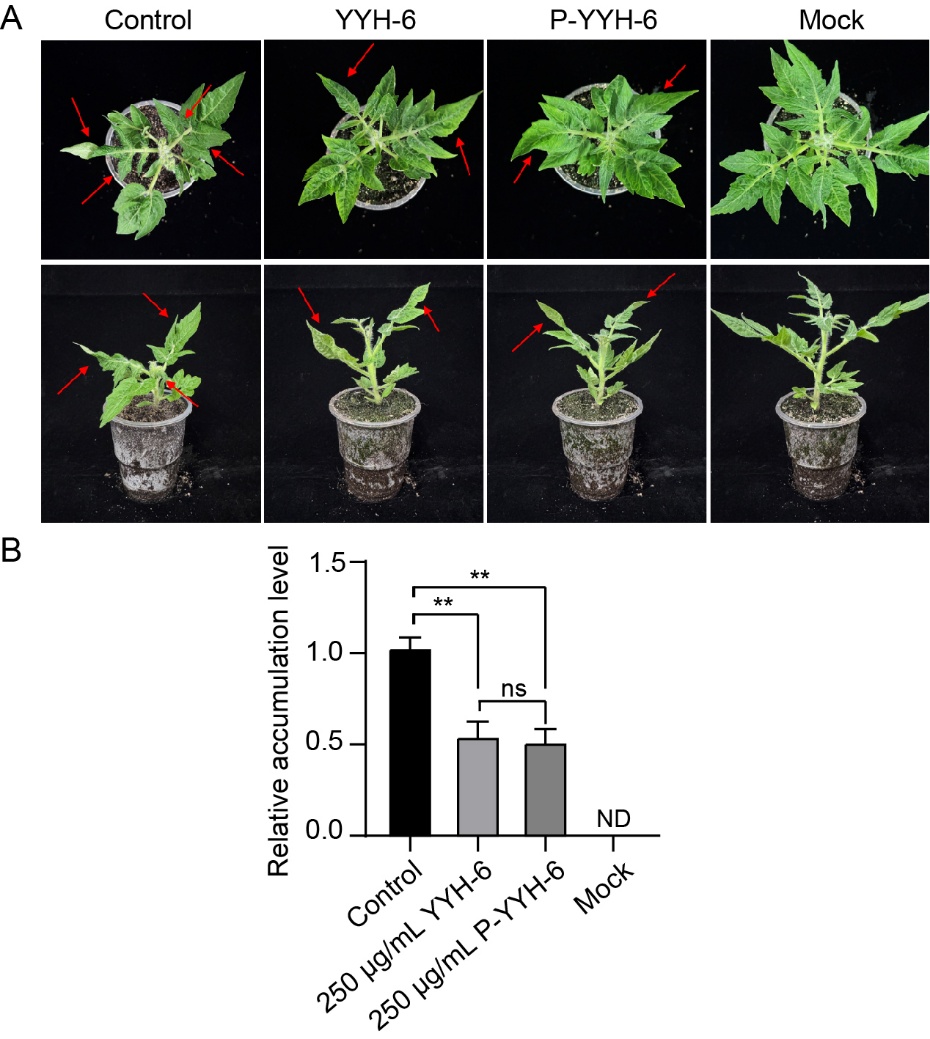


**Table S1** Nucleic acid sequences of oligonucleotide primers for vector construction.

| **Primer name** | **Sequence (5′→3′)** | **Gene symbol** |
| --- | --- | --- |
| TRV-TXNDC-F | GTGAGTAAGGTTACCGAATTCATGGCGTTAAGGCTTACACC | LOC101251929 |
| TRV-TXNDC-R | GGGACATGCCCGGGCCTCGAGGCAGCCTCGGTATCAAATCCTT |  |
| TRV-STI1CP-F | GGGACATGCCCGGGCCTCGAGGGCTTTCTTCAGTCACCTCCA | LOC101268416 |
| TRV-STI1CP-R | GTGAGTAAGGTTACCGAATTCATGGACCCGATGAAGTTGAATC |  |
| TRV-SHMT-F | GTGAGTAAGGTTACCGAATTCATGGAAGCTTGTTGTGGAGCTG | LOC101263138 |
| TRV-SHMT-R | GGGACATGCCCGGGCCTCGAGGATGTGAAATTCTCAGAGGCAA |  |
| TRV-S9POPCATCP-F | GTGAGTAAGGTTACCGAATTCATGGGTTTTTCAGGCATACCCG | LOC101255389 |
| TRV-S9POPCATCP-R | GGGACATGCCCGGGCCTCGAGCACCATACTCCTGAGCCAA |  |
| TRV-E3 RMA1H1-F | GTGAGTAAGGTTACCGAATTCGTCGTGGTAGCCCCTTA | LOC101262152 |
| TRV-E3 RMA1H1-R | CGTGAGCTCGGTACCGGATCCGCAAACAGGACATTGTGGC |  |
| TRV-RPP13-F | GTGAGTAAGGTTACCGAATTCGTAAGGGTCTTCCATTAGC | LOC101267186 |
| TRV-RPP13-R | CGTGAGCTCGGTACCGGATCCCCACCAACGGATCAGCTTGTC |  |
| TRV-ALS2-F | GTGAGTAAGGTTACCGAATTCACACACACCCAACATCATAGC | LOC101254112 |
| TRV-ALS2-R | CGTGAGCTCGGTACCGGATCCCCTGGGTATGCAAACACATCC |  |

**Table S2** Nucleic acid sequences of oligonucleotide primers for RT-qPCR.

| **Primer name** | **Sequence (5′→3′)** | **Gene symbol** |
| --- | --- | --- |
| WRKY33-F | ATCCTGCTCCGGTGCATTAC | LOC101268787 |
| WRKY33-R | GATCTTCTAGTGGATTGAGG |  |
| WRKY46-F | AGTAGGATGAGAGAGGAG | LOC101268780 |
| WRKY46-R | CTTTGGTGATGCTTCTTC |  |
| E3 RMA1H1 F | GTCGTGGTAGCCCCTTA | LOC101262152 |
| E3 RMA1H1 R | GCAAACAGGACATTGTGGC |  |
| E3 PUB22 F | AGGTTCCCTACACCTAAG | LOC101267151 |
| E3 PUB22 R | GCAACGTTTGTTAGCATC |  |
| RPP13 F | AAGTCAACACAGACAACAG | LOC101267186 |
| RPP13 R | CTCCAGTATCTCTTCAGC |  |
| ALS2 F | ATTGGGATCAGCCAATGAG | LOC101254112 |
| ALS2 R | CCACCGCATAATTAGCATAC |  |
| GST F | AGTACAGGGAGATTGGTATGG | LOC101244300 |
| GST R | CCAAATTGATCTCCTCC |  |
| LysM F | GAATATGCTGCCAATGGAC | LOC101261978 |
| LysM R | CCTTGTGAACATGAGGTG |  |
| Silence-TXNDC-F | GCAGTGACGACAACACTATC | LOC101251929 |
| Silence-TXNDC-R | GAGATGCATACTGCATTGCC |  |
| Silence-STI1CP-F | GCTGCCATACGAGATGCAAC | LOC101268416 |
| Silence-STI1CP-R | CATGCCACCAGCTCTCCTAC |  |
| Silence-SHMT-F | GAAGCATGCCAAATCACCTG | LOC101263138 |
| Silence-SHMT-R | GAGACGGACTTCTTGGCTTC |  |
| Silence-S9POPCATCP-F | GATGTGTTCAAGGCTGGAGC | LOC101255389 |
| Silence-S9POPCATCP-R | CCACATCAAAACGTCCCACC |  |
| Silence-E3 RMA1H1-F | AGCCCAACCCCTAATTTG | LOC101262152 |
| Silence-E3 RMA1H1-R | GTCCTTTTAACTGATGAT |  |
| Silence-RPP13-F | ACTGACGAACAAGTCATG | LOC101267186 |
| Silence-RPP13-R | CCAGTGTGTCCTTGCTTC |  |
| Silence-ALS2-F | ATAGCCACCACCTCA | LOC101254112 |
| Silence-ALS2-R | GGGAAGAAGGGTGGAAG |  |

**Table S7** Predicted key amino acid residues involved in the binding of YYH-6 to the target protein.

| **Proteins** | **Residues** |
| --- | --- |
| TXNDC | Leu585, Leu586, Ser588, Lys591, Arg604, Asn611, His612, Asn613, Arg614, Phe624, Leu625, Val626, Gln627, Ser630, Thr631, Gly632, Ala633, Glu634, Asn640, Phe641, Asp642, Asn659, Phe676, Val677, Glu679, Gly924, Val925, Gln926, Pro927, Pro928, Leu929, Lys930, Ser931, Arg932, Leu934, Arg936, Leu937, Arg938, Arg939, Arg940, Ser941, Gly942, Ala943, Asp944, Thr945, Lys1024, Tyr1026, Tyr1036 |
| STI1CP | Phe12, Gln15, Asn19, Ser21, Ile22, Ser24, Asp25, Pro26, Ser27, Leu28, Ala45, Asp48, Thr49, Gly50, Asp51, Tyr52, Lys53, Glu54, Lys55, Ser56, His57, Val59 |
| SHMT | Asp84, Val85, Ile88, Ile89, Asp90, Glu92, Lys93, Arg95, Gln96, Glu101, Ile103, Ser105, Glu106, Asn107, Phe108, Thr109, Val113, Met114, Val117, Gly118, His308, Lys309, Gly313, Pro314, Arg315, His348, His350, Thr351, Asp421, Thr426, Leu521, Pro522, Gly523, Leu524 |
| S9POPCATCP | Val74, Ser75, Ser77, Thr78, Arg80, Leu81, Gly82, Gly83, Phe84, Ser85, Leu95, Ser97, Pro99, Val128, Arg129, Tyr135, Gly136, Gly137, Gly138, Asp139, Phe140, Cys141, Arg339, Pro340, Trp342, Ile343, Phe344, Gly345, Met346, Lys347, Asp392, Asn394, Asn395, Asp406, Ala408, Pro413, Ser414, Ser437, Ser438, Tyr470, Arg490, His492, Gly493, Gly494, Thr496, Ala497, Glu498, Ala499, Arg500, Gly501, Cys502, Leu503, Asn504, Leu505, Thr506, Ile507, Tyr517, Asp519, Thr526, Thr569, Gly570, Ser571, Ser572, Ala573, Leu594, Tyr595, His681, Gly682, Phe683, Arg684, Ala686, Ile689, Thr692, Gln696 |
